# Semen-mediated enhancement of HIV infection is donor-dependent and correlates with the levels of SEVI

**DOI:** 10.1186/1742-4690-7-55

**Published:** 2010-06-23

**Authors:** Kyeong-Ae Kim, Maral Yolamanova, Onofrio Zirafi, Nadia R Roan, Ludger Staendker, Wolf-Georg Forssmann, Adam Burgener, Nathalie Dejucq-Rainsford, Beatrice H Hahn, George M Shaw, Warner C Greene, Frank Kirchhoff, Jan Münch

**Affiliations:** 1 Institute of Molecular Virology, University Hospital Ulm, 89081 Ulm, Germany; 2 Gladstone Institute of Virology and Immunology, University of California, San Francisco, CA 94158, USA; 3 Peptide Research Group, Clinic for Immunology, Hannover Medical School, Hannover, Germany; 4 VIRO Pharmaceuticals GmbH & Co. KG, Hannover, Germany; 5 National Laboratory for HIV Immunology, Public Health Agency of Canada, Winnipeg, Manitoba, R3E 3P6, Canada; 6 INSERM U625, Rennes; Rennes University, Groupe d'Etude de la Reproduction chez l'Homme et les Mammifères; IFR 140, Campus de Beaulieu, Rennes, France; 7 Departments of Medicine and Microbiology, University of Alabama at Birmingham, Birmingham, AL 35223, USA

## Abstract

**Background:**

HIV-1 is usually transmitted in the presence of semen. We have shown that semen boosts HIV-1 infection and contains fragments of prostatic acid phosphatase (PAP) forming amyloid aggregates termed SEVI (semen-derived enhancer of viral infection) that promote virion attachment to target cells. Despite its importance for the global spread of HIV-1, however, the effect of semen on virus infection is controversial.

**Results:**

Here, we established methods allowing the meaningful analysis of semen by minimizing its cytotoxic effects and partly recapitulating the conditions encountered during sexual HIV-1 transmission. We show that semen rapidly and effectively enhances the infectivity of HIV-1, HIV-2, and SIV. This enhancement occurs independently of the viral genotype and coreceptor tropism as well as the virus producer and target cell type. Semen-mediated enhancement of HIV-1 infection was also observed under acidic pH conditions and in the presence of vaginal fluid. We further show that the potency of semen in boosting HIV-1 infection is donor dependent and correlates with the levels of SEVI.

**Conclusions:**

Our results show that semen strongly enhances the infectivity of HIV-1 and other primate lentiviruses and that SEVI contributes to this effect. Thus, SEVI may play an important role in the sexual transmission of HIV-1 and addition of SEVI inhibitors to microbicides may improve their efficacy.

## Background

Since its introduction into the human population in the first half of the 20^th ^century by zoonotic transmission of simian immunodeficiency viruses (SIVs) found in chimpanzees [1], HIV-1 has caused one of the most devastating pandemics of modern times. To date, HIV-1 has infected more than 60 million people and caused about 25 million deaths [2]. In 2008 alone, there were 2.7 million new HIV-1 infections and 2.0 million AIDS-related fatalities. The great majority of all HIV-1 transmissions results from unprotected sexual intercourse. Despite the rapid spread of HIV-1, the estimated rate of transmission per sexual intercourse is surprisingly low: male to female virus transmission occurs about once in every 1,000-10,000 unprotected vaginal act [3,4]. Receptive anal intercourse carries an approximately 10-fold higher risk [5]. Furthermore, the rate of transmission is affected by the viral load in the donor and thus high during acute HIV-1 infection [6]. Furthermore, inflammation and lesions in the mucosal barrier resulting from other sexually transmitted diseases increase the risk of transmission [7]. Nonetheless, the dose of HIV-1 transmitted during sexual intercourse is usually subinfectious and clearly limiting viral spread [8].

Genital exposure to semen (SE) contaminated with HIV-1 accounts for most transmissions worldwide [9]. Thus, SE represents the major vector for the dissemination of HIV-1. Several intrinsic properties of SE might affect the efficiency of HIV-1 transmission, such as neutralization of the acidic pH of the vaginal fluid [10], stimulation of inflammatory cytokines [11], and induction of leukocyte infiltration of the cervical mucosa and migration of Langerhans cells [12,13]. All these effects may promote HIV-1 transmission by indirect mechanisms. It is less clear, however, whether SE directly affects the infectiousness of HIV in male genital fluid. For example, it has been reported that seminal plasma (SE-P) contains factors preventing the capture and transmission of HIV-1 to CD4+ T cells by DC-SIGN expressed on dendritic cells [14]. Another study reported that SE-P contains cationic polypeptides that inhibit HIV-1 infection [15]. On the other hand, spermatozoa themselves may capture HIV-1 through heparan sulfate and efficiently transmit the virus to dendritic cells [16].

We have previously shown that fragments of the abundant semen protein prostatic acidic phosphatase (PAP) form amyloid structures that capture HIV-1 virions and promote their attachment to target cells [17]. Strikingly, these amyloid aggregates, termed Semen-derived Enhancer of Virus Infection (SEVI), enhance the infectious titer of HIV-1 by several orders of magnitude at a low multiplicity of infection [17]. The structure of PAP248-286 (numbers refering to the amino acid position in PAP), the predominant enhancing PAP fragment detected in semen, has recently been solved and its has been confirmed that this peptide is highly amyloidogenic [18,19]. The mechanism underlying SEVI-mediated enhancement of HIV-1 infection most likely involves nucleation-dependent formation of amyloid aggregates and a direct interaction of positively charged surfaces of SEVI with negatively charged membranes [17,20,21]. SEVI seems to promote virion attachment and subsequent infection by allowing the virus to overcome the electrostatic repulsion between the negatively charged viral and cellular membranes. Notably, SEVI and SE also boost the infectiousness of XMRV (xenotropic murine leukemia virus-related virus), a novel γ-retrovirus that may be associated with prostate cancer and chronic fatigue syndrome [22] and SEVI increases the efficiency of retroviral transductions [23].

The ability of SE and SEVI to promote HIV-1 infection has been confirmed in several studies [17,20,22,24,25]. Furthermore, the first inhibitors of SE- and SEVI-mediated enhancement of HIV-1 infection have been reported [24,25] and may lead to new approaches to prevent HIV-1 transmission. Despite its possible importance in the transmissions of HIV/AIDS, however, the enhancing effect of SE on HIV-1 infection remains poorly defined and controversial. One reason is the lack of standardized methods addressing the high cytotoxicity of SE in *in vitro *culture systems. Here, we thus established methodologies allowing the meaningful analysis of SE by minimizing its cytotoxic effects. The results show that SE rapidly and effectively enhances HIV-1 infection independently of the viral strain and/or the virus producer or target cell type. Altogether, our data further support an important role of SEVI in SE-mediated enhancement of HIV-1 infection and thus in the transmission of HIV/AIDS.

## Results

### Semen boosts HIV-1 infection under non-cytotoxic conditions

**Figure 1:**
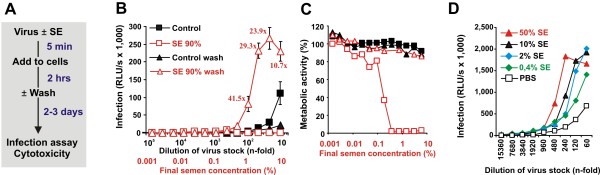
**Effect of SE on HIV infection**. (A) Schema describing the experimental procedure. (B) Effect of treatment of virus stocks with 90% (v/v) of SE on R5 HIV-1 infection. TZM-bl cells were infected with the indicated dilutions of SE- or PBS-treated virus stocks. The inoculum was either removed after 2 hours of exposure (wash) or left on the cells. Shown are average β-galactosidase activities (n = 3) measured 2 days after virus exposure. RLU/s: relative light units per second. The numbers above the upper curve give n-fold enhancement of HIV infection by SE relative to that measured for the corresponding PBS control. (C) Metabolic activities of cells analysed in B. (D) Effect of low concentrations of SE on HIV infection. R5 HIV-1 stocks were treated with the indicated concentrations of SE, diluted and used to infect TZM-bl cells. The Y-axis gives average values of triplicate infections, and the X-axis gives the final dilution of the virus stocks. The infection levels were determined as described above. Percentages refer to the SE concentrations during virion treatment. The final concentrations in the cell culture are 15-fold lower.

It is long known that the intrinsic cytotoxicity of SE complicates its meaningful analysis in cell culture [17,26]. Thus, we first established experimental conditions circumventing this problem. Specifically, we reduced the final concentrations of SE in cell culture by pre-incubating SE with the HIV-1 virions (rather than the target cells) and adding small volumes (usually 20 μl) of these HIV/SE mixtures and serial dilutions thereof, to comparatively large (typically 280 μl) TZM-bl cell cultures (Figure 1A). In some aspects this approach resembles sexual transmission of HIV, where virus-containing SE is diluted by the female genital fluid present in the vaginal tract, which has a relatively large surface area of about 100 cm^2 ^[27]. To further reduce cytotoxicity we removed the SE containing medium after 2 hours and cultured the cells in fresh medium containing gentamicin (to prevent bacterial outgrowth) until HIV-1 infection was assessed 2 to 3 days later. Under these conditions pre-treatment of virions with 90% (v/v) SE enhanced HIV-1 infection up to 40-fold compared to the untreated control (Figure 1B). In contrast, PBS-treated HIV-1 was more infectious than SE-treated virus when the inoculum was left on the target cells (Figure 1B) because final concentrations of SE as low as 1% were cytotoxic (Figure 1C). Thus, the cytotoxicity of SE may mask enhancing effects and produce misleading results, but can be overcome by reducing the concentration of SE and exposure time.

Further experiments showed that even SE concentrations as low as 0.4% during virion treatment markedly enhance HIV-1 infection (Figure 1D). Exposure to relatively high doses of SE-treated HIV-1 caused massive cytopathic effects (examples shown in Additional file 1 Figure S1). Under these conditions the detectable levels of LTR-driven reporter gene activity saturated or even declined due to over-infection and virus-mediated cell killing. Thus, HIV-1 infection rates and the effects of SE can only be faithfully determined at a relatively low multiplicity of infection. Notably, SE also enhanced infection when the HIV/SE inoculum was not removed if the size of the target cell cultures was increased to further minimize cytotoxic effects (Additional file 1 Figure S2). The ability of SE to promote HIV-1 infection was not affected by gentamicin and did not require a serum cofactor (Additional file 1 Figure S3). Furthermore, treatment with SE alone did not induce LTR-dependent reporter gene expression (data not shown).

### SE enhances HIV-1 infection rapidly and at different pH conditions

**Figure 2:**
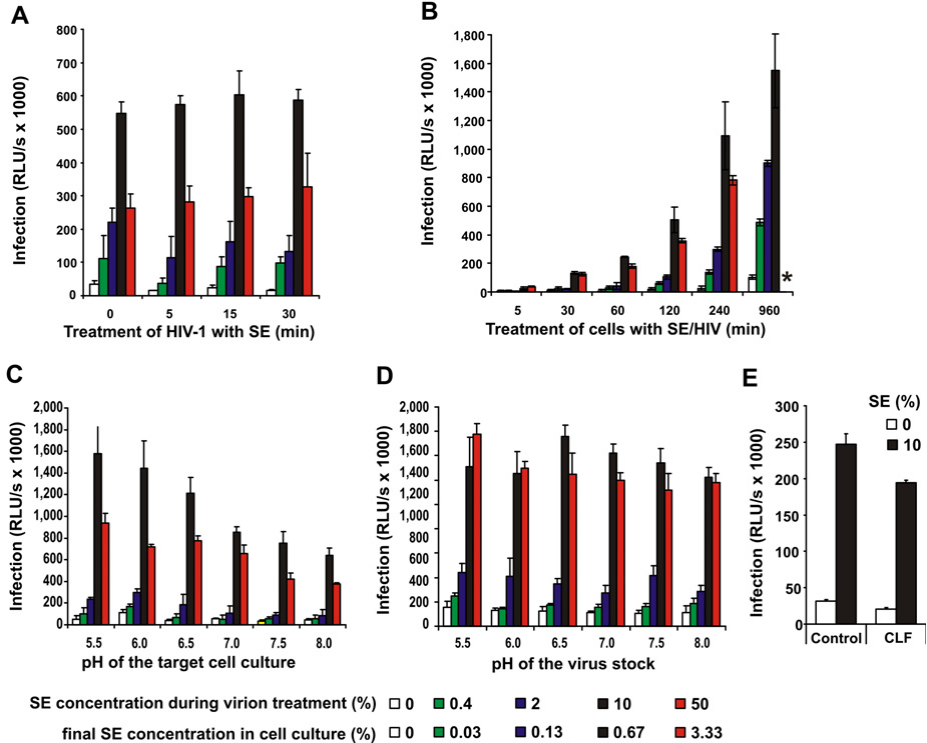
**Effect of exposure times and pH values on SE mediated enhancement of HIV infection**. (A) Effect of pre-incubation time on SE-mediated HIV infection enhancement. R5 HIV-1 was mixed with the indicated concentrations of SE, incubated for the various time periods, and 20 μl of the virus stocks was used to infect 280 μl TZM-bl cell cultures in triplicates. Values in all panels give averages ± SD (n = 3) measured 3 days after virus exposure. (B) The SE/virus mixture was incubated for 10 min at RT, and 20 μl were added to 280 μl TZM-bl cells. After different time points, the supernatant was removed, and fresh DMEM was added for further culture. The star indicates high cytotoxicity. (C) Virus stocks of R5 HIV-1 treated with the indicated concentrations of SE were used to infect TZM-bl cultures adjusted to the indicated pH values. After two hours of virus exposure, the virus stocks were removed, and the cells were cultured in fresh medium under neutral pH conditions. Higher or lower pH values could not be analyzed as they were cytotoxic. Both panels give average values ± SD (n = 3). (D) Virus stocks adjusted to the indicated pH values were either treated with PBS or with various concentrations of SE and subsequently used to infect TZM-bl indicator cells. (E) TZM-bl cells were incubated with either PBS or 10% cervico vaginal lavage (CLF) and infected with medium or 10% SE treated HIV-1. Infection rates were determined 3 dpi.

SE is composed of secretions from different sources, including the epididymis (~5% v/v of the fluid), seminal vesicles (60%), prostate (20-30%), and bulbourethral glands. The origin of seminal HIV-1 particles is still unclear [28] but they are at least in part produced within the male genital tract [29-31]. Thus, virions may be exposed to enhancing factor(s) in SE, such as fragments of PAP produced by the prostate gland, an organ productively infected by HIV/SIV [30,31], just immediately prior to their deposition in the genital tract. To assess how rapidly SE enhances the infectiousness of HIV-1, we mixed virus stocks with various concentrations of SE and used these cocktails to infect TZM-bl indicator cells after different incubation periods. We found that SE enhances HIV-1 infection, even when the HIV/SE solution was added to the cells immediately after mixing (Figure 2A). The highest levels of HIV-1 infection were usually measured after treatment with 10% SE because 50% SE (corresponding to 3.3% in the cell culture) frequently caused cytotoxic effects (Figure 2A and data not shown). To assess whether the effect of SE on HIV-1 infection depends on the duration of target cell exposure, we infected TZM-bl cells with untreated or SE-treated HIV-1 and removed the inoculum after different incubation periods. We found that the efficiency of HIV-1 infection was low during the earliest time points and increased with longer exposure times (Figure 2B). This was expected because virus entry may take several hours, and unbound or loosely attached HIV-1 virions are removed during the washing step. Importantly, SE promoted HIV-1 infection at all time points analyzed. However, the effect was most pronounced (up to 40-fold) between 1 and 4 hours of virus exposure (Figure 2B). Shorter time periods resulted in inefficient viral infection and longer incubation increased cytotoxic effects.

Next, we examined the effect of different pH conditions on SE-mediated enhancement of HIV-1 infection. This may be relevant for sexual transmission of HIV-1 because the pH in the viral environment prior to, during and after sexual intercourse may change from about 8.0 in SE to about 4.2 in the female genital fluid [10]. Unexpectedly, HIV-1 infection was moderately increased at an acidic pH in target cell cultures (Figure 2C), whereas the pH of the virus stock had no significant effect on the efficiency of HIV-1 infection (Figure 2D). Importantly, SE boosted virus infection under pH conditions ranging from 5.5 to 8.0 and in the presence of pooled cervical lavage fluid (CLF) (Figure 2E). More extreme pH conditions or increased CLF concentrations could not be analyzed, as they were toxic to the cells.

### Stability of the enhancing activity in SE

To assess the stability of the enhancing activity in SE, we incubated aliquots of pooled SE for three days at 37°C and tested its effect on HIV infection. We found that incubation of SE for 6 hours at 37°C reduced its enhancing activity by about 50% (Additional file 1 Figure S4A). Some residual activity was even detectable at 3 days of incubation (Additional file 1 Figure S4A). After sexual intercourse, elevated levels of the SE marker PAP, the precursor of SEVI, can be detected in the vagina for about 24 hours [32]. Thus, amyloidogenic PAP fragments may be generated over a period of several days. Notably, 10% SE was most effective in infectivity enhancement for the first 6 hours, whereas 50% SE showed higher activity at later time points. This indicates that the enhancing and cytotoxic factors in SE are slowly degraded. Furthermore, both activities were eliminated by heating (data not shown), suggesting that they may represent peptides or proteins. Most importantly, these data show that SE maintains some enhancing activity for several days at body temperature.

### SE generally enhances primate lentiviral infection

**Figure 3:**
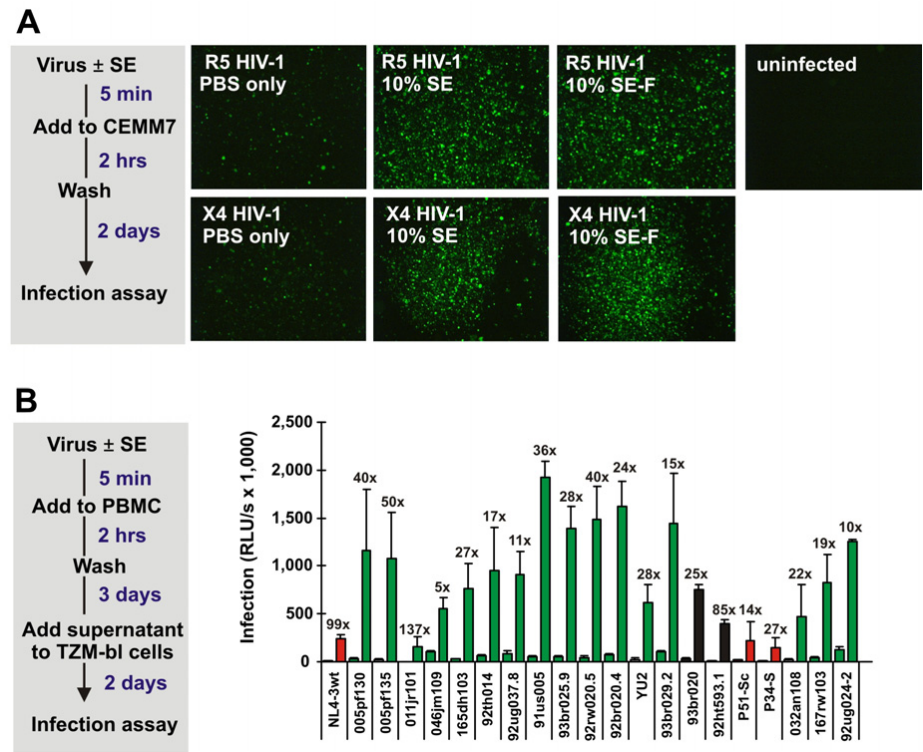
**The enhancing effect of SE is independent of the viral coreceptor tropism**. (A) Analysis of CEMx-M7 cells infected with untreated or SE-treated (10% v/v) X4 and R5 HIV-1 by fluorescence microscopy 2 dpi. (B) Treatment with SE enhances HIV-1 infection of primary PBMCs. Stimulated PBMCs were infected with the same dose of the indicated HIV-1 NL4-3 V3 recombinants that were either not treated or treated with 10% (v/v) SE. Three days later, 100 μl of the cell-free PBMC culture supernatant was used to infect TZM-bl indicator cells. Shown are average infection rates of TZM-bl cell ± SD (n = 3) measured 2 days after virus exposure. X4 virus is color-coded red; R5 virus, green; and X4R5 HIV-1, black.

All of the results described above were obtained using adherent TZM-bl cells allowing easy removal of the SE inoculum. To assess the effect of SE on HIV-1 infection in T cells, we exposed CEMx-M7 cells to SE- and SE-F-treated CXCR4(X4)- and CCR5(R5)-tropic HIV-1 strains. Examination by fluorescence microscopy and flow cytometry confirmed that treatment of HIV-1 virions with 10% SE and SE-F increased the number of GFP+ infected cells up to 17-fold (Figure 3A and Additional file 1 Figure S5A and S5B). Prior to their deposition in the genital tract, HIV-1 virions are exposed to undiluted SE. Thus, it is noteworthy that the infectiousness of HIV-1 particles was also enhanced up to 20-fold by treatment with 90% SE (Additional file 1 Figure S5C and S5D). Unexpectedly, CEMx-M7 cells infected with SE- or SE-F-treated virions also displayed about 2- to 3-fold increased levels of mean GFP expression compared to those infected with PBS-treated virus (Additional file 1 Figure S5B and S5D).

Next, we investigated whether the enhancing effect of SE on HIV-1 infection is also observed in primary human cells. To address this, we generated virus stocks of wild-type X4 HIV-1 NL4-3 and twenty V3 Envelope recombinants thereof with differential coreceptor tropism [33]. These viruses were exposed to PBS or to 10% (v/v) SE for 5 min prior to infection. A total of 20 μl of these virus stocks was then used to infect 280 μl PBMC cultures. After 3 days, the cell-free PBMC culture supernatants were used to infect TZM-bl cells (experimental outline shown in Figure 3B). To determine the effect of SE on PBMC infection and release of HIV-1 we analyzed virus production at an early time point to avoid a bias due to multiple rounds of viral replication. Strikingly, treatment with SE resulted in 5- to 137-fold (average 36.1 ± 32.7-fold, n = 21) enhancement of infectious virus in the supernatant of the PBMC cultures (Figure 3B). Effective enhancement was observed for the 16 R5 viruses as well as for the three X4 and two dual tropic HIV-1 recombinants, suggesting that SE-mediated enhancement is independent of the viral coreceptor tropism. To further examine whether SE might generally enhance primate lentiviral infection, we analyzed its effect on a large number of HIV-1, HIV-2 and SIV strains and found that SE enhanced their infectiousness by 10- to 25-fold (Additional file 1 Figure S6 and data not shown).

### SE-mediated enhancement of HIV-1 infection is independent of the virus producer and target cell type

**Figure 4:**
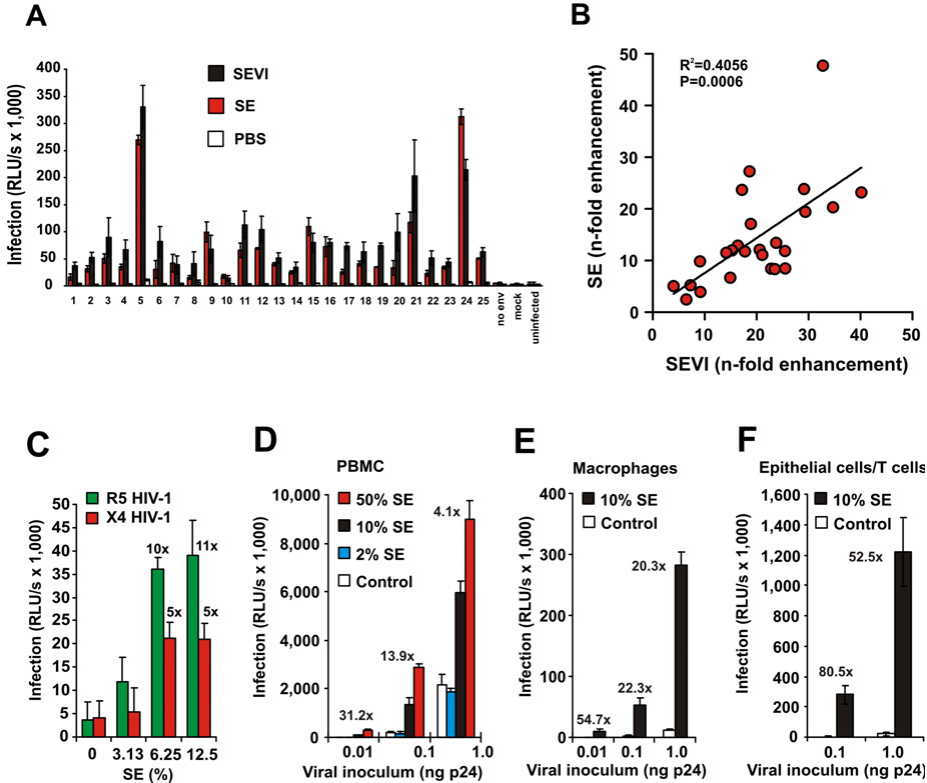
**SE enhances founder HIV infection and boosts HIV infection independently of the viral producer and target cell type**. (A) Effect of SE on HIV particles carrying gp120/41 from founder viruses. Pseudotyped HIV-1 particles were generated by transient transfection of 293T cells with an *env*-defective HIV-1 NL4-3 backbone and plasmids expressing the Env proteins previously described (34). Virions were treated with medium, 10 μg/ml SEVI or 10% SE and used to infect TZM-bl cells. The inoculum was removed after 2 hrs and infection rates were determined 2 dpi. Shown are the average levels of triplicate TZM-bl cell infections ± SD. (B) Correlation between the magnitudes of SEVI and SE-mediated enhancement of HIV-1 pseudotype infection shown in Fig 4A. N-fold enhanced infection rates were calculated by dividing infection rates obtained in the presence of SEVI or SE by those of mock-treated virus infection. (C) SE enhances infection of testis derived HIV-1. X4 tropic HIV-1 IIIb and R5 tropic SF162 were harvested from infected testis tissue, treated with indicated concentrations of SE and used to infect TZM-bl cells. (D, E) SE enhances the infectiousness of HIV-1 for PBMCs and macrophages. Stocks of an R5-tropic HIV-1 NL4-3 V3 variant (92TH04.12) containing the luciferase reporter gene in place of *nef *were generated by transient transfection of 293T cells. Virus stocks were treated with the indicated concentrations of SE and used to infect PBMC (D) and macrophages (E). Similar results were obtained using various primary HIV-1 strains. (F) SE favours *in trans*-infection of T cells by viral particles bound to epithelial cells. CaSki cells derived from an epithelial cervical carcinoma were exposed to HIV-1 treated with SE or medium for 3 hrs. Subsequently, the virus inoculum was washed out and the cells were cocultivated with CEM-M7 cells for three days. Infection rates were determined by luciferase assay. The numbers above the bars indicate n-fold enhancement relative to the infectivity measured using PBS/medium-treated virus stocks.

To assess the possible relevance of SE for sexual virus transmission, we next examined its effect on HIV-1 founder viruses, which are most likely the ones transmitted during sexual intercourse [34]. We found that SEVI and pooled SE enhance the infectiousness of HIV-1 particles pseudotyped with envelope glycoproteins derived from 25 different transmitted/founder viruses [34] in single round infection assays by 5- to 48 fold (Figure 4A). Notably, the magnitudes of infectivity enhancement by SEVI and SE correlated significantly (p = 0.0006) (Figure 4B). To further examine the effect of SE on viral particles generated by the relevant producer cells *in vivo *we harvested HIV-1 from *ex vivo *infected testis tissue [29,31]. We found that SE clearly increases the infectiousness of testis-derived R5- and X4-tropic HIV-1 strains (Figure 4C). Further experiments using luciferase reporter viruses showed that SE also promotes HIV-1 infection of primary T cells and macrophages, the relevant target cells of HIV-1 *in vivo *(Figure 4D and 4E). Finally, we examined whether SE also affects HIV-1 infection *in trans*. Our results showed that SE increases the transmission of R5-tropic HIV-1 from non-permissive CaSki human cervical epithelial carcinoma cells to susceptible T cells about 80-fold (Figure 4F). Typically, the strongest enhancing effects of SE were observed with low doses of freshly produced highly infectious HIV-1 virions, irrespective of the virus strain and producer or target cell type.

### Exploring controversies on the effect of SE on HIV-1 infection

**Figure 5:**
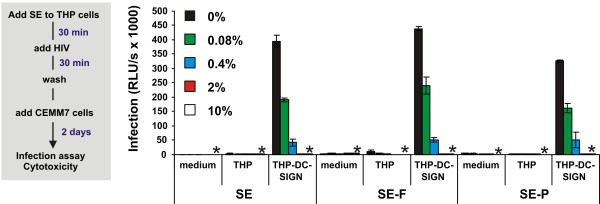
**Semen inhibits trans-infection of T cells by DC-SIGN**. B-THP-1-DC-SIGN cells were treated with the indicated concentration of SE, SE-F or SE-P for 30 min, subsequently exposed to R5 HIV-1 for 30 min, washed and cocultivated with CEM-M7 cells. The levels of infection mediated by B-THP-1 cells, which do not express DC-SIGN, and the absence of cells (medium) are also shown as controls. Shown are average values ± SD derived from triplicate infections. Stars indicate cytotoxicity, infection rates obtained after infection with 2% and 10% SE, SE-F or SE-P treated virus were close to background luciferase activities of uninfected cells.

Our result that SE enhances HIV-1 infection seems to be contradictory to previous studies reporting that seminal plasma (SE-P) impairs the capture and transmission of HIV-1 by DC-SIGN [14] and inhibits virus infection [15]. To determine the effect of SE and SE-P on HIV-1 transmission by DC-SIGN we used B-THP-1-DC-SIGN and CEM-M7 cells. As expected [14], expression of DC-SIGN strongly enhanced transmission of HIV-1 to CEM-M7 indicator cells (Figure 5). In agreement with previous results [14], pre-treatment of cells with SE, SE-F and SE-P potently inhibited DC-SIGN-mediated transmission of HIV-1 (Figure 5). In contrast, SEVI amplified infection of T cells by HIV-1 particles bound to DC-SIGN-expressing dendritic or B-THP-1 cells even further [[17], data not shown]. Thus, SEVI generally facilitates HIV-1 infection, whereas SE also contains a specific inhibitor that overcomes the enhancing effect of SEVI in the case of DC-SIGN-mediated virus transmission.

**Figure 6:**
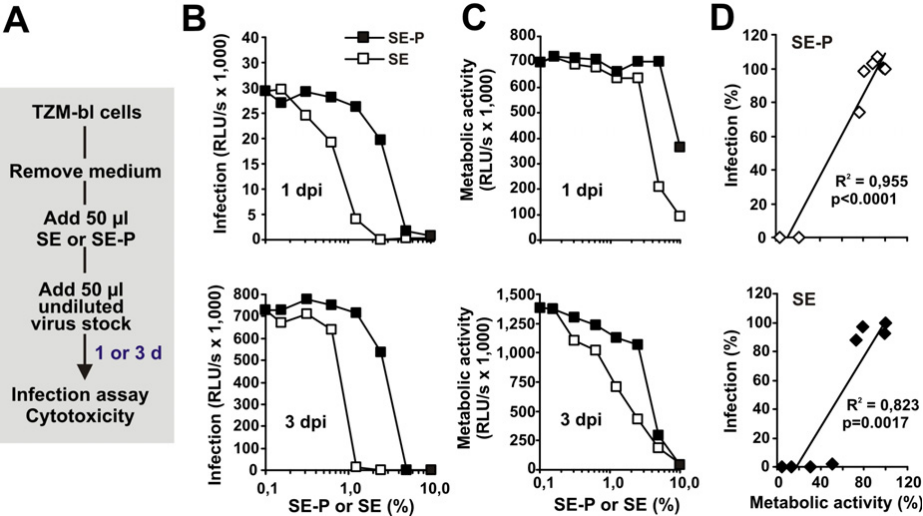
**Adding SE or SE-P directly to target cells results in reduced metabolic activity and HIV infection rates**. (A) Schematic outline of the experiment. TZM-bl cells were incubated with different dilutions of SE or SE-P and subsequently infected with HIV-1. (B) Infection rates and (C) metabolic activities were determined after 1 (upper panel) or 3 days (lower panel). (D) Correlation between Tat-driven reporter activities ("infection") and the metabolic activities of the target cells. Values were derived from the experiments shown in panels B and C, and are shown relative to those obtained in the absence of SE or SE-P (100%).

Next, we evaluated results of Martellini and coworkers suggesting that SE-P may inhibit HIV-1 [15]. In this study, the target cells and not the HIV-1 virions were treated with SE-P. To assess the effect of SE on the susceptibility of target cells to HIV-1 infection, we used a flow cytometry-based HIV-1 virion-fusion assay [35]. This system allows to directly measure virion fusion with the target cells and minimizes cytotoxic effects because the cells are only exposed to SE for short time periods. Primary endometrial CD4^+ ^T cells were either PBS-treated or treated with SE, washed, exposed to HIV-1 NL4-3 BlaM-Vpr virions for 4 hours and loaded with CCF2/AM dye. In agreement with published data [20,24], treatment with 10% SE enhanced the susceptibility of the cells to HIV-1 infection by 5-fold (Additional file 1 Figure S7). This effect is lower than that observed in HIV-1 infection assays because the fusion assay requires higher viral doses. Nonetheless, our data show that treatment with SE enhances rather than reduces the susceptibility of cells to HIV-1 infection. To elucidate why Martellini and coworkers obtained different results, we repeated the experiments following their protocols (Figure 6A). In agreement with their findings, treatment of the TZM-bl indicator cells with SE-P and SE and subsequent infection with HIV-1 resulted in reduced levels of Tat-driven reporter gene activity suggesting inhibition of virus infection (Figure 6B). At one day post-infection (the time point examined in the previous study) cytotoxic effects were observed at about 4-fold higher concentrations of SE-P and SE compared to those required to inhibit virus infection (Figure 6C, upper). At 3 days post-infection cytotoxic effects become more apparent (Figure 6C, lower) and the metabolic activity of the cells correlated significantly with the Tat-driven reporter gene activities (Figure 6D). Thus, a decreased metabolic activity of the target cells rather than a specific anti-HIV activity of SE-P or SE may account for the reduced levels of Tat-driven reporter gene activity in this assay. Efficient viral gene expression is dependent on the "fitness" of the cells and is highly sensitive to cytotoxic or cytostatic effects and experimental conditions just at the threshold of cytotoxicity may yield misleading results.

### SE-mediated enhancement of HIV-1 infection is donor-dependent and correlates with the levels of PAP248-286/SEVI

**Figure 7:**
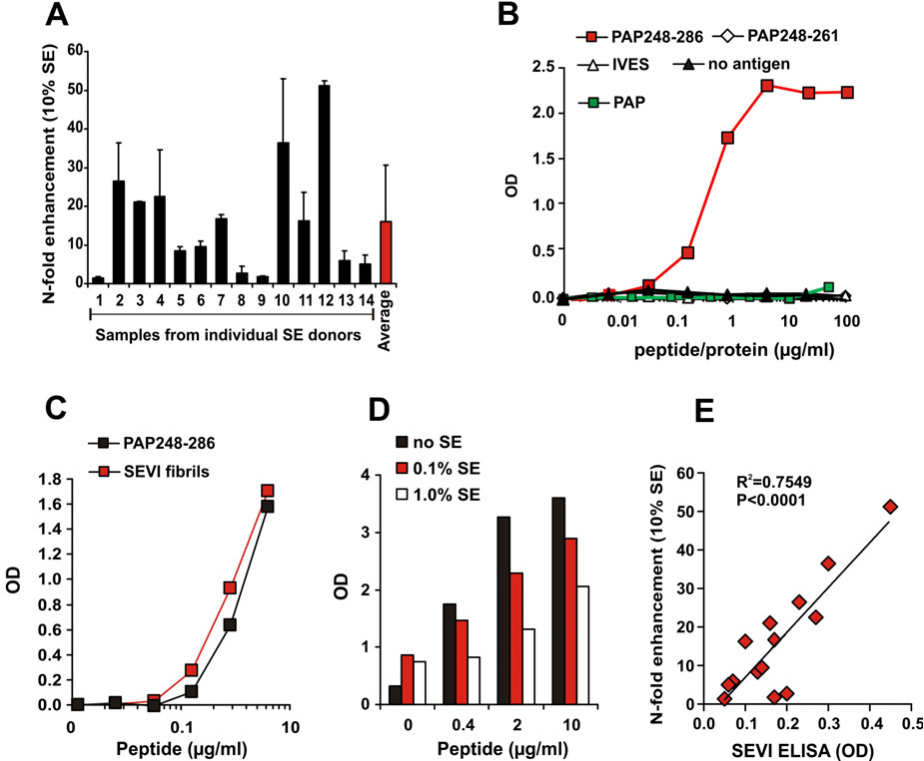
**The HIV enhancing activity of individual SE samples correlates with SEVI concentrations**. (A) Enhancement of HIV-1 infection by SE samples from 14 different donors. R5 HIV-1 stocks were mixed with 10% (v/v) of the SE samples or PBS and used for infection of TZM-bl indicator cells. Similar results were obtained using SE samples from more than 80 additional donors. Reactivity of anti-SEVI-antiserum from guinea pigs to (B) the indicated monomeric peptides or IVES, a peptide containing the reverse amino acid sequence of PAP248-286 and full-length PAP. (C) SEVI fibrils or (D) pooled SE spiked with SEVI. Similar results were obtained using an antiserum from rabbits. (E) Correlation between the magnitude of enhancement of HIV-1 infection by individual SE samples and the quantity of SEVI/PAP248-286 detectable using the anti-SEVI antiserum.

All experiments described above were performed with pooled semen obtained from more than 20 individual donors. To assess whether the enhancing activity may be donor-dependent, we collected and analyzed SE samples from 14 different individuals. All SE samples were allowed to liquefy for 30 min and subsequently kept frozen until further use. Notably, they were processed and tested together for their ability to promote HIV-1 infection. We found that all SE samples enhanced HIV-1 infection, albeit with strikingly different efficiencies ranging from 2- to about 50-fold (Figure 7A). Thus, in addition to the viral load and other factors, the potency of SE in enhancing the infectiousness of HIV-1 particles may also affect the rates of virus transmission. Notably, fresh liquefied ejaculates enhanced HIV-1 infection about as effectively as SE samples that had undergone a freeze/thaw cycle (data not shown). Thus, the presence of living spermatozoa capturing virions by heparan sulfate [16] or other cells did not interfere with SE-mediated enhancement of HIV infection.

To assess a possible role of SEVI in the differential capacity of these SE samples to enhance HIV-1 infection, we generated specific antibodies by immunizing rabbits and guinea pigs with amyloidogenic PAP248-286. Both immune sera (but not the pre-immune sera) reacted with monomeric PAP248-286 and with SEVI amyloid, but not with shorter PAP fragments (e.g. PAP248-261), a peptide containing the reverse amino acid sequence of PAP248-286 (termed "IVES"), or with full-length PAP (Figure 7B and 7C, and data not shown). Importantly, the sera also recognized SEVI spiked into SE (Figure 7D) and allowed to establish a semi-quantitative indirect "SEVI ELISA". The potency of the 14 SE samples described above in enhancing HIV-1 infection correlated significantly with their reactivity to the anti-SEVI sera (Figure 7E). This finding was confirmed with a large number of SE samples from both Germany and the US (data not shown). Notably, centrifugation of SE-F through a 100-kDa-pore-size filter removed the entire virus enhancing activity and the reactivity to anti-SEVI antiserum (Additional file 1 Figures S4B and S4C). Thus, the enhancing factor in SE has a molecular weight of > 100 kDa, which is in agreement with the large size of amyloid aggregates. Altogether, these data are further evidence that SEVI contributes to SE-mediated enhancement of HIV-1 infection. Furthermore, they emphasize the importance of using pooled SE samples for a valid analysis of SE-mediated enhancement of HIV-1 infection because the activity of individual samples varies substantially.

## Discussion

SE is the main vector for the spread of the AIDS pandemic [9] and contains cell-free HIV-1 virions, even in individuals receiving HAART [36,37]. It is well established that the levels of infectious virus transmitted during sexual intercourse are a major determinant of the rate of HIV-1 transmission [6,38]. Nonetheless, strikingly little is known about the effect of SE on the infectiousness of HIV-1. Our results suggest that the ability of SE to boost HIV-1 infection may have been missed because its meaningful analysis is not without complications. We show that the confounding effects of cytotoxic factors in SE can largely be overcome by (i) pre-treating the virus rather than the cells; (ii) using a small volume of SE-treated virus stocks to infect a large target cell culture and (iii) removing the HIV/SE inoculum after a few hours. While the analysis of SE for enhancement of HIV-1 infection requires some optimization, these conditions actually partly recapitulate those encountered during sexual transmission of HIV-1. Other treatments commonly used to examine SE, such as pre-incubation of target cell, heat treatment and infection with high concentrations of freeze-thawed viral inoculums, all reduce the ability of SE to enhance HIV-1 infection, or may even misleadingly suggest that it is inhibitory.

The newly established experimental conditions allowed to demonstrate that SE promotes HIV-1 infection independently of the viral geno- and phenotype or the viral producer and target cell type. Importantly, our analyses included HIV-1 founder viruses as well as the primary cell types relevant for virus production and infection *in vivo *(Figure 4A). The enhancing effect of SE was relatively stable (Additional file 1 Figure S4) and observed over a broad range of pH conditions as well as in the presence of vaginal fluid (Figure 2C-E). Our finding that SE increases the infectiousness of HIV-1 almost instantaneously (Figure 2A) suggests that it may also affect the rate of female-to-male virus transmission, particularly if mixtures of HIV-1 containing vaginal fluid and semen become stuck under the foreskin. Notably, the observed effects most likely underestimate the potency of SE in boosting HIV-1 infection because its cytotoxicity precluded the analysis of high SE concentrations or long cellular exposure times.

Our previous screening of a highly complex protein/peptide library from pooled SE identified only enhancing amyloidogenic peptides, but no inhibitors of HIV [17]. However, we also confirmed findings of Sabatte and coworkers [14] showing that pretreatment of DC-SIGN expressing cells with SE inhibits the transmission of HIV-1 to CD4+ target cells. This inhibitor could not be detected in our screening approach because its molecular mass exceeds the cut-off size used for the generation of the SE-derived peptide/protein library and because we examined effects on CD4/coreceptor-mediated HIV-1 infection and not on the interaction of the virus with DC-SIGN. The evidence that HIV-1 does not effectively bind to DC-SIGN in the presence of SE argues against a major role of this C-type lectin in sexual transmission of HIV-1. However, it is conceivable that a potent attachment factor like SEVI may allow HIV-1 to bypass the requirement for cellular attachment factors. In agreement with this possibility, SEVI and SE drastically enhance HIV-1 infection of T cells both directly and *in trans *by epithelial cells (Figure 4) [17]. Thus, amyloid aggregates in SE may help the virus at the earliest stage of infection, when it is most vulnerable to elimination [8], by promoting both virion binding to protrusions of DCs extending to the luminal surface and by enhancing virus infection of CD4+ target cells that become accessible through physical breaks in the epithelial barrier.

We have previously shown that the small precipitate obtained after centrifugation of SE-P contains HIV enhancing activity and a high quantity of amyloidogenic PAP248-286 [17]. Here, we show that the potency of individual SE samples in enhancing HIV-1 infection correlates with levels of SEVI (Figure 7E). This result further supports that SEVI contributes to the enhancing effect of SE on HIV-1 infection. Additional experiments are required to fully elucidate the underlying mechanism. However, all data are consistent with the possibility that amyloid aggregates bind to both the virions and the cells, thereby allowing them to overcome the repulsion between their negatively charged membrane surfaces [20]. Relatively small amyloid aggregates like oligomers or filaments with sizes in the lower nanometer range may account for the bulk of the enhancing activity because large fibrils (> 0.1 micrometer) are not detectable in SE [17]. Indeed, it has been suggested that small fibrils are particularly active in enhancing HIV-1 infection [39].

We found that every SE sample that liquefied and thus could be analyzed enhanced HIV-1 infection. However, our results also showed that the potency of SE in enhancing the infectiousness of HIV-1 virions varies between different donors. It has been previously shown that some HIV-1-infected individuals transmit the virus more efficiently than others [40,41]. Based on the evidence that a small subset of infected people may be responsible for a disproportionately high number of HIV-1 transmission, it has been suggested that "super-spreaders" may play a significant role in the spread of the AIDS pandemic [42]. Our findings suggest that in addition to the viral load present in the genital fluid, the differential potency of SE to enhance the infectiousness of HIV-1 virions may play a relevant role in the rate of virus transmission occurring after unprotected sexual intercourse.

Although our data are highly suggestive, the importance of SE and SEVI for the spread of HIV-1 *in vivo *remains elusive. Previous studies in the SIV/macaque model yielded somewhat contradictory data. Neildez and colleagues observed that treating a low dose of SIVmac251 with SE-P increased vaginal transmission rates [43], whereas others failed to detect significant effects of SE or SE-P on SIV transmission [44]. However, the latter may be due to the fact that the animal numbers were low and the experimental conditions did not reflect the *in vivo *situation, e.g., (i) the virus doses used were several orders of magnitude higher than those usually transferred during sexual intercourse; (ii) frozen virus stocks that may contain a relatively high proportion of defective particles were used for infection and (iii) frozen SE-P was administered into the vagina prior to the inoculation with SIV. We found that SE and SEVI efficiently promote SIV infection *in vitro *and we are planning to perform low-dose vaginal challenge studies in the SIV/macaque model [45] to assess the effect of SE on virus transmission *in vivo*.

Although SE may strongly enhance the infectiousness of HIV, the rate of sexual virus transmission per unprotected vaginal intercourse is low. It is conceivable that even an effective attachment factor will not allow the virus to penetrate an intact mucosal surface. Further experimentation is necessary, but it is tempting to speculate that amyloid aggregates in SE increase the stickiness of HIV-1 virions, thereby increasing the chances of the virus attaching to and infecting target cells that may become accessible through microlesions or local inflammation. SE itself may play a role in the recruitment of target cells to the site of primary virus exposure by stimulating inflammatory cytokines that may induce transient infiltration of the cervical mucosa by leucocytes and the migration of Langerhans cells [2,13,46].

The fact that SE enhances the infectiousness of HIV-1 may have important implications for the development of preventive vaccines and microbicides. It is still not fully understood whether HIV-1 transmission by sexual intercourse usually results from cell-free or cell-associated virus. However, a recent study analyzing the origin of HIV-1 strains among men who have sex with men provided phylogenetic evidence that seminal plasma virus is the source [47]. Thus, it is conceivable that blocking SE's ability to enhance HIV-1 infection may reduce virus transmission and the first inhibitors of SEVI have already been identified [24,25,48]. SE-mediated enhancement of HIV-1 infection may also affect the antiviral potency of antibodies, microbicides and antiretroviral agents. In fact, recent studies suggest that seminal plasma reduces the sensitivity of HIV-1 to inhibition by microbicides [49,50], which may explain why clinical trials have thus far generally failed [51].

## Conclusions

SE efficiently enhances HIV-1 infection independently of the virus strain and producer or target cell type. The magnitude of enhancement is donor-dependent and correlates with the levels of SEVI. The enhancing effect of SE on HIV-1 infection should be considered in the development of preventive agents and the inhibition of this host enhancing activity may help to reduce the rates of sexual transmission of HIV/AIDS.

## Methods

### SE and SE-F

Semen samples were collected from healthy individuals with informed consent. Individual SE samples were obtained from the "Kinderwunschzentrum" (Goettingen, Germany) or the UCSF Fertility Clinic (San Francisco, USA). Pooled SE was generated from SE samples derived from > 20 individual donors. All ejaculates were allowed to liquefy for 30 min and SE samples were stored in 1 ml aliquots at -20°C. SE-F represents the cell free supernatant of SE pelleted for 5 min at 10000 rpm. In all experiments, aliquots were rapidly defrosted, analyzed immediately and the remainder discarded.

### HIV-1 variants and virus stocks

Virus stocks of NL4-3-derived V3 recombinants [29], HIV-1 NL4-3_R5_G-Luc (encoding the secretable *Gaussia princeps *luciferase in place of *nef*), HIV-1 virions pseudotyped with the envelope glycoproteins from different transmitted/founder viruses [34], HIV-2 ROD10 (kindly provided by K. Strebel), HIV-2 7312 (kindly provided by G. Shaw and B.H. Hahn), HIV-1 SG3.1, BRU/LAI and YU2 (obtained through the NIH AIDS Research and Reference Reagent Program) and SIVmac239 were generated by transient transfection of 293T cells as described [17]. After overnight incubation, the transfection mixture was replaced by 2 ml DMEM medium supplemented with 10% FCS, and the cells were cultured for 36 hours. Subsequently, the culture supernatant was centrifuged for 5 min at 3,000 rpm to remove cell debris. The resulting virus stock was analyzed by p24 antigen ELISA. Virus stocks were either used immediately or stored in aliquots at -80°C. Testis-derived virus was obtained at day 14 post-infection of testis explants infected with HIV-1 SF162 and IIIB as described [29].

### Cell culture

TZM-bl cells (obtained through the NIH ARRRP from Dr. John C. Kappes, Dr. Xiaoyun Wu and Tranzyme, Inc.) and CEMxM7 cells (kindly provided by N. Landau) were cultured as previously described [17]. Human PBMC were obtained by ficoll density centrifugation and CD14-CD4+ cells were isolated by magnetic bead separation (Miltenyi). 5 × 10^5 ^cells were seeded in RPMI medium (10% FCS, Pen/Strep, Glu,) in 96-well round-bottom plates and pre-treated with SE for one hour.

### Effect of SE on HIV-1 infection of TZM-bl cells

Standard testing of the effect of SE on HIV infection was performed using adherent TZM-bl reporter cells. Typically, 1 × 10^4 ^cells were seeded in microtiter plates in a volume of 280 μl medium (DMEM supplemented with 10% FCS, 100 units/mL penicillin, 100 μg/mL streptomycin and 50 μg/mL gentamicin). The following day, frozen SE samples were thawed, briefly vortexed, diluted in Dulbecco's PBS (1×) or medium, and mixed 1/1 (v/v) with virus stocks. In most experiments, SE was serially diluted 5-fold resulting in 100%, 20%, 4% and 0.8% solutions. Subsequently, 40 μl of these dilutions were transferred to U-bottom microtiter plates and mixed with 40 μl of undiluted, 10-fold and 100-fold diluted virus stocks (corresponding to 2.0, 0.2 and 0.02 ng p24 antigen); referred to as "virion treatment". Dilutions of the virus stock were used to avoid over-infection of the target cells in the presence of SE. The concentrations of SE during virion treatment were thus 50%, 10%, 2%, 0.4% and 0%. After 1 to 5 min incubation at RT, the HIV/SE mixtures were resuspended twice, and 20 μl thereof were used to infect 280 μl TZM-bl cells (70% confluent) resulting in a 15-fold dilution of SE and thus final SE concentrations in the cell culture of 3.3%, 0.66%, 0.13%, 0.03% and 0%. Final SE concentrations > 0.1% were cytotoxic when left on the cells for longer time periods. To avoid this, the HIV/SE inoculum was removed after 2 hrs of incubation at 37°C and the cells were further cultivated in 200 μl fresh DMEM (supplemented with 10% FCS and antibiotics). Even under these conditions, concentrations of ≥3.3% SE reduced the viability of the cells. Infection was monitored daily by light microscopy and infection rates were determined upon occurrence of virus induced cytopathic effects, or after a maximum of 3 days. Infection rates of TZM-bl were determined using the one-step Tropix Gal-Screen Kit (Applied Biosystems) as recommended by the manufacturer. β-galactosidase activities were determined using the Orion microplate luminometer (Berthold). All values represent reporter gene activities (relative light units per second; RLU/s) derived from triplicate infections minus background activities derived from uninfected cells. To analyze the effect of SE on HIV infection under conditions approximating the in vivo situation, 20 μl of virus stocks were mixed with 180 μl SE (corresponding to 90% SE during virion incubation). Subsequently, 2-fold serial dilutions in PBS or medium were generated and 20 μl aliquots thereof were used to infect 280 μl TZM-bl cell cultures. The HIV/SE inoculum was removed 2 hrs later and infection rates were determined as described above.

### Effect of SE on HIV infection of PBMCs, macrophages and CEMx-M7 cells

PBMC were isolated and cultivated as described [17]. Virus stocks were treated with 10% SE or PBS, and 20 μl of the HIV/SE mixtures were added to 280 μl PBMCs (total of 2 × 10^5 ^cells), resulting in a final SE concentration of 0.66%. After 3 hrs, the cells were transferred to V-shaped microtiter plates and pelleted at 1,000 rpm for 5 min. Supernatants were removed and cells resuspended in 200 μl fresh medium. At 3 days post-infection, 100 μl of the PBMC supernatants were used to infect 100 μl TZM-bl cell cultures (1 × 10^4 ^cells). Two days later, infection rates were determined as described above. To monitor the effect of SE on HIV infection by fluorescence microscopy, a total of 2 × 10^5 ^CEMx174 M7 cells containing the GFP reporter gene under the control of the HIV-1 promoter were seeded in 280 μl medium and infected with 20 μl of 10% SE-treated HIV-1. After 2 hrs cells were pelleted and resuspended in fresh medium. Aliquots of the cultures were analyzed by UV microscopy 3 days later. Macrophages were generated by treating buffy coat-derived monocytes for 3 days with GMCSF (10 ng/ml) followed by MCSF (2 ng/ml) for another 3 days. Cells were then seeded at a density of 20,000 cells/well in 280 μl RPMI containing MCSF and infected with SE-treated or mock-treated HIV-1 NL4-3_R5_G-Luc. Two hrs later, the cells were washed to remove Gaussia luciferase introduced by the virus stock and 250 μl fresh medium was added. At 3 days post-infection, 40 μl of the culture supernatant and 40 μl of the washing control were analyzed for Gaussia luciferase activity using the Gaussia-Juice Kit (P.J.K) as recommended by the manufacturer. Luciferase activities were determined using the Orion microplate luminometer (Berthold). Reporter activities in all controls were <1,000 RLU/s.

### Effect of SE on HIV transmission from CaSki cells to T cells

10,000 CaSki cells (ATCC #CRL-1550), an epithelial cervical carcinoma cell line, were seeded in 280 μl medium and infected the next day with 20 μl of SE- or mock-treated HIV. After 3 hrs, the inoculum was removed and CaSki cells were co-cultivated with 5 × 10^4 ^CEMx-M7 cells in 200 μl medium. Three days later, luciferase activities in cellular lysates were determined using the luciferase assay system (Promega).

### Cell Viability

The effect of SE on metabolic activity of cells was analyzed using the MTT assay under experimental conditions corresponding to those used in the respective infection assays. After 3 days of incubation, 20 μl of a 5 mg/ml MTT (3-(4,5-dimethylthiazole-2-yl)-2,5-diphenyl tetrazolium bromide, Sigma #M2003) solution was added to cells. After 3 hrs, the cell-free supernatant was discarded, formazan crystals were dissolved in 100 μl DMSO-ethanol (1:1) and OD was detected at 490/650 nM. In some experiments cytotoxic effects of SE were further analyzed using the CellTiter-Glo Luminescent Cell Viability Assay (Promega, #G7571) as recommended by the manufacturer.

### Effect of SE on DC-SIGN-mediated *trans*-HIV infection

Seminal plasma (SE-P) was prepared by centrifugation of pooled SE for 30 min at 1,000 g. The supernatant was passed through 0.22-μm-pore-size filters. 20 μl SE or SE-P and 5-fold dilutions thereof were added to 160 μl THP-1 or THP-DC-SIGN cells (2 × 10^5^) or medium only. After 30 min incubation at 37°C, 20 μl R5 HIV-1 (undiluted, 10-, 100- and 1,000-fold diluted) was added and incubated for another 30 min. Thereafter, cells were transferred into 96-well V-shape plates, pelleted by centrifugation, washed two times in PBS to remove unbound virus and resuspended in 100 μl medium. Subsequently, 100 μl CEMx-M7 cells (1 × 10^5^) were added. After 3 days of co-cultivation, 150 μl of the cultures were pelleted, and luciferase activities in the cell lysates were detected using Promega's luciferase assay kit as recommended by the manufacturer.

### HIV-1 infection of cells pretreated with SE or SE-P

The experiment was performed essentially as described [15] except that virus stocks containing normalized quantities (0.5 ng of p24 antigen) of an R5 tropic HIV-1 NL4-3 variant were used and that infection rates and metabolic activities were measured not only after 1 but also after 3 days post-infection. SE-P was generated by pelleting SE for 30 min at 1,500 g [15]. TZM-bl cells (6 × 10^4^) were seeded the day before infection. The next day 50 μl of serial dilutions of SE and SE-P were added and cells were infected with 50 μl virus stocks containing 0.5 ng p24. One and three days later, infection rates and cellular ATP levels as indicator of metabolic activity were determined using the Promega luciferase assay kit or CellTiter-Glo Luminescent Cell Viability Assay. We also examined the ATP levels of SE and SE-P themselves in the absence of cells under the same experimental conditions.

### SEVI ELISA

96 well EIA/RIA plates (Corning Incorporated) were coated with 100 μl dilutions of antigen or 100-fold dilutions of cell free SE-F in PBS over night at 4°C. The next day, plates were rinsed twice in wash buffer (imidazole-buffered Saline with Tween 20; KPL), blocked for 2 hrs with 100 μl 1 × Roti^®^-Block solution (Roth) and rinsed again 5 times. Thereafter, 100 μl of 50-fold dilutions of pre-immune sera or antisera derived from SEVI amyloid immunized New Zealand White female rabbits (d28; Pocono Rabbit Farms) or guinea pigs (S3; IPF-Pharmaceuticals) were added, and then plates were incubated for 1 hr and rinsed 5 times. Finally, 100 μl of 10,000 fold dilutions of HRP-coupled anti-rabbit (PerkinElmer) or anti-guinea-pig antibodies (Abcam) were added, samples were incubated for 30 min, washed 5 times, and incubated with 100 μl HRP substrate (SureBlue™, KPL). Reactions were stopped by adding 100 μl 1 N HCl and OD was read at 450/650 nm. Correlation analyses were performed using GraphPad Prism software.

### SE-F filtration

500 μl SE-F was filtered through a Microcon YM-100 Centrifugal Filter Unit (Millipore, #42424). The retentate and filtrate were used immediately in the standard TZM-bl infection assay.

### Effect of different pH conditions

To assess whether the pH of the target cell culture affects SE-mediated enhancement of HIV-1 infection, we replaced the TZM-bl culture medium by 280 μl DMEM adjusted to various pH values with acetic acid, HCl or NaOH, just prior to infection. At 2 hrs after infection, this medium was replaced by standard DMEM. To examine whether the pH value of the virus stock impacts the enhancing effect of SE, virus stocks were diluted 10-fold in DMEM adjusted to different pH values and subsequently treated with SE and analyzed in infection assays as described above.

### Effect of SEVI on HIV pseudotype infection

PAP248-286 obtained from VIRO Pharmaceuticals GmbH & Co KG (Hannover, Germany) was dissolved in PBS (10 mg/ml), and 200 μl aliquots were agitated at 1,300 rpm overnight at 37°C in 1.5 ml microtubes using a thermomixer. SEVI amyloid formation was verified by staining with CongoRed and Thioflavin T as described [17]. 40 μl of virus stocks were treated with 40 μl of a 20 μg/ml SEVI dilution or 20% SE or medium for 5 min, and 20 μl of mixtures were used to infect TZM-bl cells. Infection rates were determined 3 days post-infection as described above.

### HIV-1 Virion-Fusion

This flow cytometry-based HIV-1 virion-fusion assay was conducted as previously described [17,20]. This assay is based on the incorporation of beta-lactamase-Vpr (BlaM-Vpr) chimeric proteins into HIV-1 virions and their subsequent delivery into target cells loaded with the CCF2 fluorescent dye by virion fusion. In infected cells, BlaM cleaves CCF2 and changes the fluorescence from green to blue, thereby allowing detection of fusion by flow cytometric analysis.

## Competing interests

The authors declare that they have no competing interests.

## Authors' contributions

KAK and MY performed most of the experiments, OZ and NRR established the SEVI ELISA and NRR performed the fusion assay, LS and WGF synthesized PAP248-286 and generated SEVI antibodies, AB performed CVL experiments, NDR generated testis derived virus, BHH, GMS and WCG provided reagents, FK and JM conceived and coordinated the study and wrote the final manuscript. All authors read and approved the final manuscript.

## Supplementary Material

Additional File 1 **Supplementary figures**. Click here for file

## References

[B1] Korber B Muldoon M Theiler J Gao F Gupta R Lapedes A Hahn BH Wolinsky S Bhattacharya T Timing the ancestor of the HIV-1 pandemic strains Science 2000 288 1789 96 10.1126/science.288.5472.1789 10846155

[B2] UNAIDS. 2008 AIDS epidemic update http://www.unaids.org

[B3] Gray RH Wawer MJ Brookmeyer R Sewankambo NK Serwadda D Wabwire-Mangen F Lutalo T Li X vanCott T Quinn TC Rakai Project Team Probability of HIV-1 transmission per coital act in monogamous, heterosexual, HIV-1-discordant couples in Rakai, Uganda Lancet 2001 357 1149 53 10.1016/S0140-6736(00)04331-2 11323041

[B4] Padian NS Shiboski SC Glass SO Vittinghoff E Heterosexual transmission of human immunodeficiency virus (HIV) in northern California: results from a ten-year study Am J Epidemiol 1997 146 350 57 9270414 10.1093/oxfordjournals.aje.a009276

[B5] Vittinghoff E Douglas J Judson F McKirnan D MacQueen K Buchbinder S Per-contact risk of human immunodeficiency virus transmission between male sexual partners Am J Epidemiol 1999 150 306 11 10430236 10.1093/oxfordjournals.aje.a010003

[B6] Pilcher CD Tien HC Eron JJ Jr Vernazza PL Leu SY Stewart PW Goh LE Cohen MS Quest Study; Duke-UNC-Emory Acute HIV Consortium Brief but efficient: acute HIV infection and the sexual transmission of HIV J Infect Dis 2004 189 1785 92 10.1086/386333 15122514

[B7] Galvin SR Cohen MS The role of sexually transmitted diseases in HIV transmission Nat Rev Microbiol 2004 2 33 42 10.1038/nrmicro794 15035007

[B8] Haase AT Perils at mucosal front lines for HIV and SIV and their hosts Nat Rev Immunol 2005 5 783 92 16200081 10.1038/nri1706

[B9] Royce RA Seña A Cates W Jr Cohen MS Sexual transmission of HIV N Engl J Med 1997 336 1072 78 10.1056/NEJM199704103361507 9091805

[B10] Tevi-Bénissan C Bélec L Lévy M Schneider-Fauveau V Si Mohamed A Hallouin MC Matta M Grésenguet G In vivo semen-associated pH neutralization of cervicovaginal secretions Clin Diagn Lab Immunol 1997 4 367 74 9144379 10.1128/cdli.4.3.367-374.1997 PMC170534

[B11] Sharkey DJ Macpherson AM Tremellen KP Robertson SA Seminal plasma differentially regulates inflammatory cytokine gene expression in human cervical and vaginal epithelial cells Mol Hum Reprod 2007 13 491 501 10.1093/molehr/gam028 17483528

[B12] Berlier W Cremel M Hamzeh H Lévy R Lucht F Bourlet T Pozzetto B Delézay O Seminal plasma promotes the attraction of Langerhans cells via the secretion of CCL20 by vaginal epithelial cells: involvement in the sexual transmission of HIV Hum Reprod 2006 21 1135 42 10.1093/humrep/dei496 16531471

[B13] Thompson LA Barratt CL Bolton AE Cooke ID The leukocytic reaction of the human uterine cervix Am J Reprod Immunol 1992 28 85 89 1285856 10.1111/j.1600-0897.1992.tb00765.x

[B14] Sabatté J Ceballos A Raiden S Vermeulen M Nahmod K Maggini J Salamone G Salomón H Amigorena S Geffner J Human seminal plasma abrogates the capture and transmission of human immunodeficiency virus type 1 to CD4+ T cells mediated by DC-SIGN J Virol 2007 81 13723 34 10.1128/JVI.01079-07 17913809 PMC2168832

[B15] Martellini JA Cole AL Venkataraman N Quinn GA Svoboda P Gangrade BK Pohl J Sørensen OE Cole AM Cationic polypeptides contribute to the anti-HIV-1 activity of human seminal plasma FASEB J 2009 23 3609 18 10.1096/fj.09-131961 19487309 PMC3236594

[B16] Ceballos A Remes Lenicov F Sabatté J Rodríguez Rodrígues C Cabrini M Jancic C Raiden S Donaldson M Agustín Pasqualini R Marin-Briggiler C Vazquez-Levin M Capani F Amigorena S Geffner J Spermatozoa capture HIV-1 through heparan sulfate and efficiently transmit the virus to dendritic cells J Exp Med 2009 206 2717 33 10.1084/jem.20091579 19858326 PMC2806607

[B17] Münch J Rücker E Ständker L Adermann K Goffinet C Schindler M Wildum S Chinnadurai R Rajan D Specht A Giménez-Gallego G Sánchez PC Fowler DM Koulov A Kelly JW Mothes W Grivel JC Margolis L Keppler OT Forssmann WG Kirchhoff F Semen-derived amyloid fibrils drastically enhance HIV infection. Cell 2007 131 1059 1071 10.1016/j.cell.2007.02.042 18083097

[B18] Nanga RP Brender JR Vivekanandan S Popovych N Ramamoorthy A NMR structure in a membrane environment reveals putative amyloidogenic regions of the SEVI precursor peptide PAP(248-286) J Am Chem Soc 2009 131 17972 79 10.1021/ja908170s 19995078 PMC2792124

[B19] Brender JR Hartman K Gottler LM Cavitt ME Youngstrom DW Ramamoorthy A Helical conformation of the SEVI precursor peptide PAP248-286, a dramatic enhancer of HIV infectivity, promotes lipid aggregation and fusion Biophys J 2009 97 2474 83 10.1016/j.bpj.2009.08.034 19883590 PMC2770606

[B20] Roan NR Münch J Arhel N Mothes W Neidleman J Kobayashi A Smith-McCune K Kirchhoff F Greene WC The cationic properties of SEVI underlie its ability to enhance human immunodeficiency virus infection J Virol 2009 83 73 80 10.1128/JVI.01366-08 18945786 PMC2612336

[B21] Ye Z French KC Popova LA Lednev IK Lopez MM Makhatadze GI Mechanism of fibril formation by a 39-residue peptide (PAPf39) from human prostatic acidic phosphatase Biochemistry 2009 48 11582 91 10.1021/bi901709j 19902966

[B22] Hong S Klein EA Das Gupta J Hanke K Weight CJ Nguyen C Gaughan C Kim KA Bannert N Kirchhoff F Munch J Silverman RH Fibrils of prostatic acid phosphatase fragments boost infections with XMRV (xenotropic murine leukemia virus-related virus), a human retrovirus associated with prostate cancer J Virol 2009 83 6995 7003 10.1128/JVI.00268-09 19403677 PMC2704761

[B23] Wurm M Schambach A Lindemann D Hanenberg H Ständker L Forssmann WG Blasczyk R Horn PA The influence of semen-derived enhancer of virus infection on the efficiency of retroviral gene transfer J Gene Med 2010 12 137 46 20052742 10.1002/jgm.1429

[B24] Roan NR Sowinski S Münch J Kirchhoff F Greene WC Aminoquinoline surfen inhibits the action of SEVI (semen-derived enhancer of viral infection) J Biol Chem 2010 285 1861 69 10.1074/jbc.M109.066167 19897482 PMC2804344

[B25] Hauber I Hohenberg H Holstermann B Hunstein W Hauber J The main green tea polyphenol epigallocatechin-3-gallate counteracts semen-mediated enhancement of HIV infection Proc Natl Acad Sci USA 2009 106 9033 38 10.1073/pnas.0811827106 19451623 PMC2683882

[B26] Allen RD Roberts TK The relationship between the immunosuppressive and cytotoxic effects of human seminal plasma Am J Reprod Immunol Microbiol 1986 11 59 64 3740349 10.1111/j.1600-0897.1986.tb00030.x

[B27] Pendergrass PB Belovicz MW Reeves CA Surface area of the human vagina as measured from vinyl polysiloxane casts Gynecol Obstet Invest 2003 55 110 13 10.1159/000070184 12771458

[B28] Le Tortorec A Dejucq-Rainsford N HIV infection of the male genital tract - consequences for sexual transmission and reproduction Int J Androl 2009 19531082 10.1111/j.1365-2605.2009.00973.x PMC2816356

[B29] Roulet V Satie AP Ruffault A Le Tortorec A Denis H Guist'hau O Patard JJ Rioux-Leclerq N Gicquel J Jégou B Dejucq-Rainsford N Susceptibility of human testis to human immunodeficiency virus-1 infection in situ and in vitro Am J Pathol 2006 169 2094 103 10.2353/ajpath.2006.060191 17148672 PMC1762481

[B30] Le Tortorec A Le Grand R Denis H Satie AP Mannioui K Roques P Maillard A Daniels S Jégou B Dejucq-Rainsford N Infection of semen-producing organs by SIV during the acute and chronic stages of the disease PLoS One 2008 3 3 e1792 10.1371/journal.pone.0001792 18347738 PMC2268241

[B31] Le Tortorec A Satie AP Denis H Rioux-Leclercq N Havard L Ruffault A Jégou B Dejucq-Rainsford N Human prostate supports more efficient replication of HIV-1 R5 than X4 strains ex vivo Retrovirology 2008 5 119 10.1186/1742-4690-5-119 19117522 PMC2649003

[B32] Collins KA Bennett AT Persistence of spermatozoa and prostatic acid phosphatase in specimens from deceased individuals during varied postmortem intervals Am J Forensic Med Pathol 2001 22 228 32 10.1097/00000433-200109000-00004 11563728

[B33] Papkalla A Münch J Otto C Kirchhoff F Nef enhances human immunodeficiency virus type 1 infectivity and replication independently of viral coreceptor tropism J Virol 2002 76 8455 59 10.1128/JVI.76.16.8455-8459.2002 12134048 PMC155138

[B34] Keele BF Giorgi EE Salazar-Gonzalez JF Decker JM Pham KT Salazar MG Sun C Grayson T Wang S Li H Wei X Jiang C Kirchherr JL Gao F Anderson JA Ping LH Swanstrom R Tomaras GD Blattner WA Goepfert PA Kilby JM Saag MS Delwart EL Busch MP Cohen MS Montefiori DC Haynes BF Gaschen B Athreya GS Lee HY Wood N Seoighe C Perelson AS Bhattacharya T Korber BT Hahn BH Shaw GM Identification and characterization of transmitted and early founder virus envelopes in primary HIV-1 infection Proc Natl Acad Sci USA 2008 105 7552 57 10.1073/pnas.0802203105 18490657 PMC2387184

[B35] Cavrois M De Noronha C Greene WC A sensitive and specific enzyme-based assay detecting HIV-1 virion fusion in primary T lymphocytes Nat Biotechnol 2002 20 1151 54 10.1038/nbt745 12355096

[B36] Gupta P Mellors J Kingsley L Riddler S Singh MK Schreiber S Cronin M Rinaldo CR High viral load in semen of human immunodeficiency virus type 1-infected men at all stages of disease and its reduction by therapy with protease and nonnucleoside reverse transcriptase inhibitors J Virol 1997 71 6271 75 9223532 10.1128/jvi.71.8.6271-6275.1997 PMC191898

[B37] Zhang H Dornadula G Beumont M Livornese L Jr Van Uitert B Henning K Pomerantz RJ Human immunodeficiency virus type 1 in the semen of men receiving highly active antiretroviral therapy N Engl J Med 1998 339 1803 9 10.1056/NEJM199812173392502 9854115

[B38] Chakraborty H Sen PK Helms RW Vernazza PL Fiscus SA Eron JJ Patterson BK Coombs RW Krieger JN Cohen MS Viral burden in genital secretions determines male-to-female sexual transmission of HIV-1: a probabilistic empiric model AIDS 2001 15 621 27 10.1097/00002030-200103300-00012 11317000

[B39] Wojtowicz WM Farzan M Joyal JL Carter K Babcock GJ Israel DI Sodroski J Mirzabekov T Stimulation of enveloped virus infection by beta-amyloid fibrils J Biol Chem 2002 277 35019 24 10.1074/jbc.M203518200 12119288

[B40] Clumeck N Taelman H Hermans P Piot P Schoumacher M De Wit S A cluster of HIV infection among heterosexual people without apparent risk factors N Engl J Med 1989 321 1460 62 2811959 10.1056/NEJM198911233212107

[B41] Peterman TA Stoneburner RL Allen JR Jaffe HW Curran JW Risk of human immunodeficiency virus transmission from heterosexual adults with transfusion-associated infections JAMA 1988 259 55 58 10.1001/jama.259.1.55 3334772

[B42] Hyman JM Li J Stanley EA Modeling the impact of random screening and contact tracing in reducing the spread of HIV Math Biosci 2003 181 17 54 10.1016/S0025-5564(02)00128-1 12421551

[B43] Neildez O Le Grand R Chéret A Caufour P Vaslin B Matheux F Théodoro F Roques P Dormont D Variation in virological parameters and antibody responses in macaques after atraumatic vaginal exposure to a pathogenic primary isolate of SIVmac251 Res Virol 1998 149 53 68 10.1016/S0923-2516(97)86900-2 9561564

[B44] Miller CJ Marthas M Torten J Alexander NJ Moore JP Doncel GF Hendrickx AG Intravaginal inoculation of rhesus macaques with cell-free simian immunodeficiency virus results in persistent or transient viremia J Virol 1994 68 6391 400 8083977 10.1128/jvi.68.10.6391-6400.1994 PMC237059

[B45] Regoes RR Longini IM Feinberg MB Staprans SI Preclinical assessment of HIV vaccines and microbicides by repeated low-dose virus challenges PLoS Med 2005 2 e249 10.1371/journal.pmed.0020249 16018721 PMC1176242

[B46] Sharkey DJ Macpherson AM Tremellen KP Robertson SA Seminal plasma differentially regulates inflammatory cytokine gene expression in human cervical and vaginal epithelial cells Mol Hum Reprod 2007 13 491 501 10.1093/molehr/gam028 17483528

[B47] Butler DM Delport W Kosakovsky Pond SL Lakdawala MK Man Cheng P Little SJ Richman DD Smith DM The Origins of Sexually Transmitted HIV Among Men Who Have Sex with Men Sci Transl Med 2010 2 18re1 20371483 10.1126/scitranslmed.3000447 PMC2945226

[B48] Schuksz M Fuster MM Brown JR Crawford BE Ditto DP Lawrence R Glass CA Wang L Tor Y Esko JD Surfen, a small molecule antagonist of heparan sulfate Proc Natl Acad Sci USA 2008 105 13075 80 10.1073/pnas.0805862105 18725627 PMC2529023

[B49] Neurath AR Strick N Li YY Role of seminal plasma in the anti-HIV-1 activity of candidate microbicides BMC Infect Dis 2006 6 150 10.1186/1471-2334-6-150 17042959 PMC1618840

[B50] Patel S Hazrati E Cheshenko N Galen B Yang H Guzman E Wang R Herold BC Keller MJ Seminal plasma reduces the effectiveness of topical polyanionic microbicides J Infect Dis 2007 196 1394 402 10.1086/522606 17922405

[B51] Morris GC Lacey CJ Microbicides and HIV prevention: lessons from the past, looking to the future Curr Opin Infect Dis 2010 23 57 63 10.1097/QCO.0b013e328334de6d 19918175

