# Supplementary material for: Semen-mediated enhancement of HIV infection is donor-dependent and correlates with the levels of SEVI
Source: Retrovirology. 2010 Jun 23;7:55. doi: 10.1186/1742-4690-7-55 (PMC2914040; doi:10.1186/1742-4690-7-55)
Supplement: Additional File 1 — Supplementary figures. [file 1742-4690-7-55-S1.PDF]

Semen-mediated enhancement of HIV infection is donor-dependent and correlates with the levels of SEVI

Kyeong-Ae Kim, Maral Yolamanova, Onofrio Zirafi, Nadia R. Roan, Ludger Staendker, Wolf-Georg Forssmann, Adam Burgener, Nathalie Dejucq-Rainsford, Beatrice H. Hahn, George M. Shaw, Warner C. Greene, Frank Kirchhoff, and Jan Münch

SUPPLEMENTARY FIGURES:

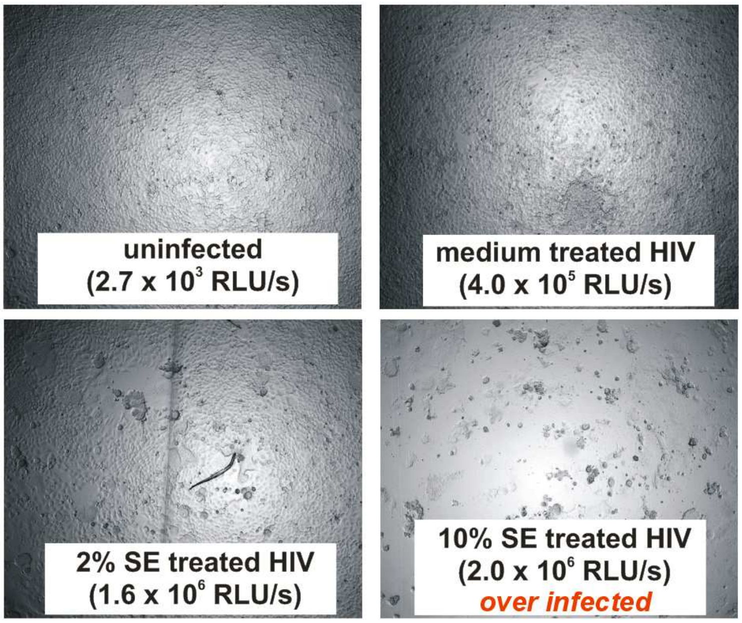

Supplementary Fig. 1. Microscopic examination of TZM-bl cells that were left uninfected or exposed to equal doses of HIV-1 either treated with PBS or the indicated concentrations of SE. Numbers give ß-galactosidase activities in the cellular extracts 2 days post infection. Similar results were obtained in numerous independent experiments.

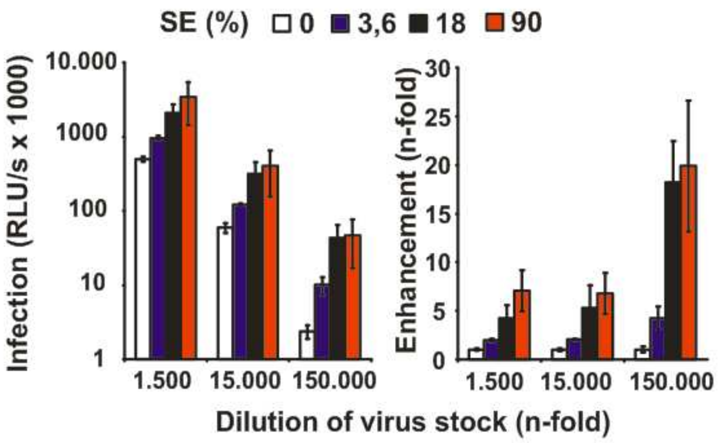

Supplementary Fig. 2. At high final dilutions SE enhances HIV infection when the HIV/SE inoculum is left on the cells. Undiluted or 1:10 and 1:100 diluted R5 HIV-1 stocks were treated with the indicated concentrations of SE for 10 min. Subsequently, 20µl of these virus stocks were used to infect 2980 µl TZM-bl cell cultures. Infection rates were determined 3 days post infection. The X-axis indicates the final dilution of the virus stocks. Shown are average values ±SD derived from triplicate infections. Similar results were obtained in an independent experiments and using SE-F.

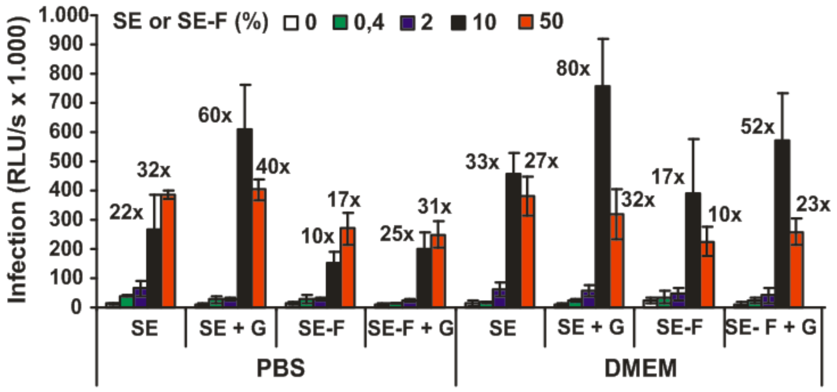

Supplementary Fig. 3. SE-mediated infectivity enhancement is not affected by gentamicin (G) or fetal calf serum. TZM-bl cells were exposed to R5 HIV-1 treated with the indicated concentrations of SE diluted either in PBS or in FCS-free DMEM for 4 hrs and subsequently cultured in fresh DMEM supplemented with 10% FCS in the presence of absence of gentamicin. After two days, infectivity was detected by measuring ß-galactosidase activities in cellular lysates. Shown are average values derived from triplicate infections +/- SD. Numbers indicate n-fold infectivity enhancement observed in the presence of the indicated concentrations of SE or SE-F.

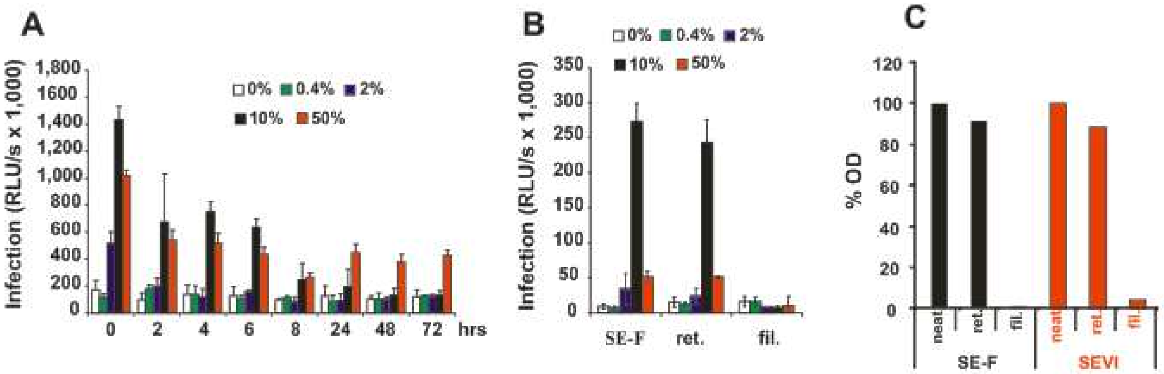

Supplementary Fig. 4. Stability and characteristics of the enhancing factor in SE. (A) Stability of the enhancing activity in SE at body temperature. R5 HIV-1 stocks were mixed with aliquots of pooled SE incubated at 37 °C for the indicated time periods and subsequently used to infect TZM-bl indicator cells. (B) The MW of the enhancing factor is larger than 100 kDa. SE-F was centrifuged through filters with 100 kDa pore size. The original solution, the retentate (ret.) and the filtrate (fil.) were diluted, incubated with HIV-1 and used to infect TZM-bl cells and ß-galactosidase activities determined 3 days later. (C) SE-F and SEVI were centrifuged through filters with 100 kDa pore size and SEVI concentrations were determined by ELISA. Values represent OD values relative to non-centrifugated SE-F or SEVI controls, respectively.

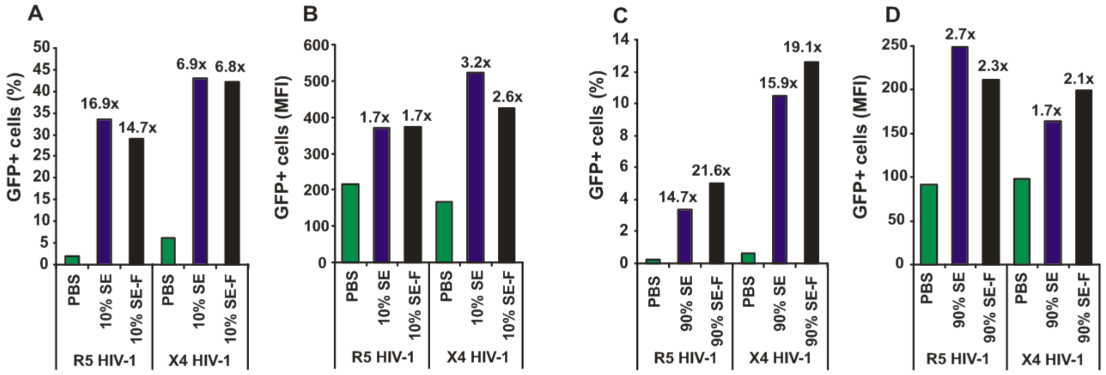

Supplementary Fig. 5. Effect of SE on X4 and R5 HIV-1 infection of CEMx-M7 cells.

The cell cultures shown in Fig. 4A of the main text were analyzed by flow cytometric analysis for the percentages of GFP+ HIV-1-infected cells (A) and the mean fluorescence intensities of GFP expression (B). The numbers above the bars indicate n-fold enhancement by SE treatment. (C, D) Effect of treatment with 90% (v/v) SE on R5 and X4 HIV-1 infection of CEMx-M7 cells. The absolute levels of GFP+ HIV-1-infected cells are lower than in panel B because a 5-fold lower viral dose was used for infection.

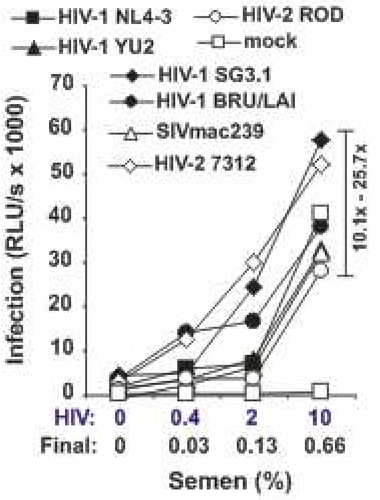

Supplementary Fig. 6. SE enhances HIV-1, HIV-2 and SIV infection. 293T-derived virus stocks were treated for 5 min with the indicated SE concentrations. Subsequently, these virus stocks were used to infect TZM-bl cells in triplicate. The inoculum was removed after 2 hrs, fresh medium added and infection rates determined 3 dpi. The X-axis indicates the percentage of SE during virus incubation (blue) and the final concentration in the cell culture (black).

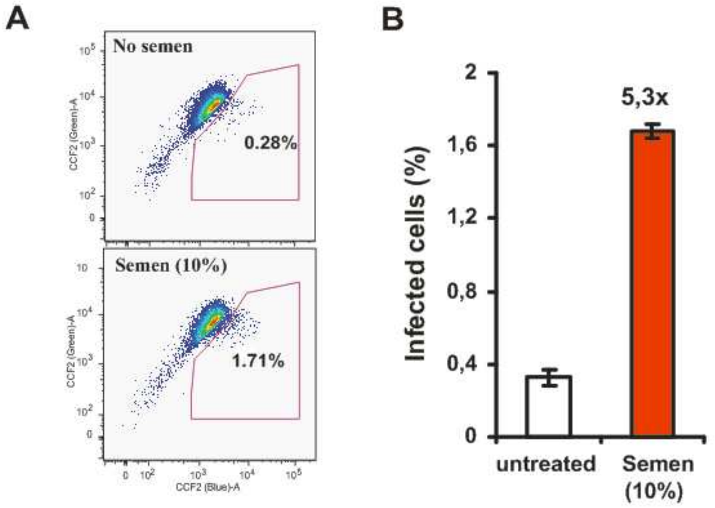

Supplementary Fig. 7. Treatment of target cells with SE increases fusion with HIV-1 virions. Untreated or SE-treated (10% v/v) primary endometrial CD4+ T cells were infected with the same dose of HIV-1 NL4-3 BlaM-Vpr virions. (A) Shows representative FACS dot plots for the detection of CCF2 substrate cleavage in CD4+ T cells which were either not treated or treated with SE prior to infection. (B) Average percentages (n=5) of HIV-1-infected untreated or SE-treated target cells.
